# Supplementary material for: Variability in sodium content of takeaway foods: Implications for public health and nutrition policy
Source: PLoS One. 2026 Jan 21;21(1):e0339339. doi: 10.1371/journal.pone.0339339 (PMC12822929; doi:10.1371/journal.pone.0339339)
Supplement: Attachment — Submitted filename: kuhnle_reply.pdf [file pone.0339339.s001.pdf]

# PONE-D-25-12820 Variability in Sodium Content of Takeaway Foods: Implications for Public Health and Nutrition Policy

Dear Dr Nyarko:

Thank you for considering our paper “Variability in Sodium Content of Takeaway Foods: Implications for Public Health and Nutrition Policy” for publication in the “PLOS One”. We are very grateful for the time the reviewers have taken to provide their extensive and constructive feedback.

We address their points individually below and indicate the changes we have made in response.

Yours sincerely

Gunter G. C. Kuhnle

---

## Reviewer 1

**Point 1.1**: Please clarify why the failure of food databases to capture the high degree of variablity in nutrient content is particularly important for sodium (page 1, last sentence).

**Response**: Thank you for this suggestion. We have revised the sentence as follows: *his is particularly problematic for nutrients like sodium, which is frequently added during food preparation in variable amounts, often without precise measurement. Content can therefore differ substantially between otherwise similar products. Because sodium is a key modifiable risk factor for hypertension and cardiovascular disease, even small discrepancies between labelled and actual values can have significant implications for both individual health and population-level intake estimates.*

**Point 1.2**: Please discuss the adverse health effects of an excessive sodium intake in greater detail.

**Response**: Thank you for this suggestion, we have added the following paragraph: *Elevated sodium consumption is causally linked to increased blood pressure, which in turn raises the risk of stroke, coronary heart disease, and other cardiovascular conditions. Meta-analyses of randomised controlled trials have shown that modest, sustained reductions in sodium intake can lead to significant decreases in systolic and diastolic blood pressure across diverse populations. Estimates by the World Health Organization suggest that excess sodium intake contributes to up to 1.8 million deaths annually, making it one of the leading dietary risk factors for mortality and disease burden globally. These risks are particularly relevant for vulnerable populations, including those with hypertension, kidney disease, or other non-communicable conditions. Therefore, improving the accuracy of sodium content information is essential for effective dietary guidance and policy intervention.*

**Point 1.3**: Figure 2 is interesting but I believe a Bland-Altman Difference Plot would capture your findings in a more adequate way.

**Response**: Thank you for the suggestion. We prepared a Bland–Altman plot and explored several visualisations in that style. However, we found that these did not provide additional information beyond what is already presented in our current figures. Our study compares a measured sodium content against a declared value, which functions as a target or policy estimate rather than a measurement, which is outside of the scope of these plots. Moreover, because our sample is not statistically representative, the visual impression of systematic bias in such a plot could be misleading. We therefore opted not to include it in the manuscript.

---

## Reviewer 2

**Point 2.1**: I am sincerely missing which is the first aim of this manuscript as, from one side authors seem to evidence the difference in the labelled sodium content vs the analyzed, but then they are also claiming – starting from the title – a public health and nutrition policy aim.

**Response**: Thank you for pointing this out. The primary aim of the study, as explained in the introduction, is to evaluate the discrepancy between labelled and measured sodium content. Sodium is one of the key target for public health interventions, and therefore, these discrepancies have implications for both dietary surveillance and policy. Public health interventions should acknowledge the variability in food composition and ensure that this is communicated also to the consumer. As we have outlines in the conclusion, "menu labels should not be considered definitive but rather as general indicators of sodium content and other nutrients."

**Point 2.2**: First of all, the "takeaway" product category has no scientific meaning or definition. So, each product which is prepared somewhere for selling and kept away from that place can be takeaway. Of course in takeaway there can be present products not packaged, and so authors cannot compare the sodium content.

**Response**: "Takeaway" is used frequently in the scientific literature, although we agree that it is not a precisely defined scientific category. We have added an explanatory footnote to provide a more detailed explanation.

**Point 2.3**: Then, in table 1 there are some "cherry picked" products with different recipes, which can differ from each other also in the sale from different markets.

**Response**: We strongly disagree with the suggestion that products were *cherry picked*. This implies intentional selection bias, which is not supported by our methodology. As stated in the manuscript, food items were chosen to reflect the variety of commonly available takeaway meals in Reading, UK. The selection process was random within categories, and we made a deliberate effort to include multiple outlets and brands (including chain and independent vendors). For each item, multiple samples were collected to account for intra-outlet variation. Importantly, we do not claim that our sample is representative of all takeaway foods in the UK, nor do we generalise our findings beyond the observed range. Rather, our aim was to characterise the variability that consumers may encounter in real-world settings. This is explicitly acknowledged in the limitations section, and the conclusions are framed accordingly.

**Point 2.4**: Then, I cannot understand which is the public health and nutrition policy. Authors are not referring to documents which set benchmarks for the "takeaway" products, i.e. https://www.who.int/publications/i/item/9789240092013, and are not comparing such values with the reference.

**Response**: We have now included references to the *WHO global sodium benchmarks for different food categories* and the UK's *Sugar, salt and calorie reduction and reformulation* policy.

**Point 2.5**: More, authors are not suggesting what it can be done to reduce the salt and reformulate the product (if it can be reformulated).

**Response**: Reformulation of products is beyond the scope of this manuscript.
