# Supplementary material for: Variability in sodium content of takeaway foods: Implications for public health and nutrition policy
Source: PLoS One. 2026 Jan 21;21(1):e0339339. doi: 10.1371/journal.pone.0339339 (PMC12822929; doi:10.1371/journal.pone.0339339)
Supplement: Attachment — Submitted filename: kuhnle_reply_v2.pdf [file pone.0339339.s002.pdf]

# PONE-D-25-12820R01 Variability in Sodium Content of Takeaway Foods: Implications for Public Health and Nutrition Policy

Dear Dr Nyarko:

Thank you for considering our paper "Variability in Sodium Content of Takeaway Foods: Implications for Public Health and Nutrition Policy" for publication in the "PLOS One". We are very grateful for the time the reviewers have taken to provide their extensive and constructive feedback.

We address the point made by reviewer 2 below:

Yours sincerely

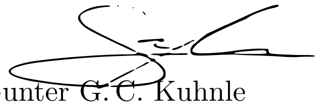

Gunter G. C. Kuhnle

**Point 0.1**: The revision of the authors substantially not considered any of the aspect rose up by the referee, including the comparison with WHO benchmark/

**Response**: We appreciate the reviewer's suggestion regarding WHO benchmarks. While these global reference values are important, compliance with UK salt-reduction targets was more relevant for this study, as they are the operational standards guiding product reformulation in the UK. To avoid confusion between policy levels, WHO values were mentioned only in the Discussion.

**Point 0.2**: an explication of the selection of the products, which of course cannot be a reference for UK product, but such product have no real selection method.

**Response**: The product selection process has now been clarified (Methods): "All foods (n=3 per item, see Table 1) were purchased from a random selection of takeaway outlets across Reading in the County of Royal Berkshire (England, United Kingdom), to capture the diversity of foods typically purchased locally. "
