# Supplementary material for: Variability in sodium content of takeaway foods: Implications for public health and nutrition policy
Source: PLoS One. 2026 Jan 21;21(1):e0339339. doi: 10.1371/journal.pone.0339339 (PMC12822929; doi:10.1371/journal.pone.0339339)
Supplement: Attachment — Submitted filename: kuhnle_reply_v3.pdf [file pone.0339339.s003.pdf]

PONE-D-25-12820R02 Variability in Sodium Content of Takeaway Foods: Implications for Public Health and Nutrition Policy

Dear Raposo:

Thank you for considering our paper “Variability in Sodium Content of Takeaway Foods: Implications for Public Health and Nutrition Policy” for publication in the “PLOS One”. We are very grateful for the time the reviewers have taken to provide their extensive and constructive feedback.

We address the point made by reviewers 2 and 4 below:

Yours sincerely

Gunter G. C. Kuhnle

Reviewer 2

Point 0.1: Concerning the first comment, I cannot see any WHO benchmark related mention in conclusion, and also references in the text are not available.
Concerning the second comment "a random selection of the products" cannot be a proof of "to capture the diversity of foods typically purchased locally. " In fact, being random chosen, it cannot be covered the differences in terms of salt neither in terms of diffusion of the products neither in terms of consumption.

Response: We agree that a simple random purchase cannot guarantee representativeness of local dietary patterns. Our intention was narrower: to avoid subjective selection of outlets or products and to reflect what is available for purchase in the study area. This has been made clear throughout the text and we never claim that this sample is representative.
We have added the following paragraph to the discussion:
This study focuses on the UK, where salt-reduction initiatives and product-specific benchmarks have been in place for many years [UK Benchmark]. Although our sample is small and not designed to be representative of all UK foods, it illustrates the degree of compositional variability that is likely to occur more widely, given that production processes are broadly similar across settings. A larger, country-specific investigation would be required to assess compliance with international WHO benchmarks [WHO Benchmark].

Reviewer 4

Point 0.2: I found your work to be highly relevant, well-structured, and significantly important in addressing the challenges associated with dietary sodium intake and menu labeling accuracy in takeaway foods.

Response: We would like to thank Reviewer 4 for the kind words.

Point 0.3: there is slight discrepancy in your recommendation in Abstract and the main document- "Regular validation of menu labels and improved consumer guidance are needed" this did not appear in the main document but in manuscript.

Response: Thank you for pointing this out. In order to align with the main conclusion of the study — i.e. that content labels on food items should be seen more as indicative and not definitive amount — we have changed the abstract and replaced the last sentence with "It is therefore important that menu labels are not considered definitive but rather general indicators of sodium content and potentially other nutrients."

Point 0.4: Thee World Health Organization suggest that excess sodium intake contributes to up to 1.8 million deaths annually, making it one of the leading dietary risk factors for mortality and disease 23 burden globally) it is good that you have glimpsed from global perspective and it would be good if relate and connect to the global salt reduction target

Response: Thank you for this comment, and we appreciate the importance of recognising international benchmarks. However, as the UK has a dedicated salt-reduction campaign with specific benchmarks, we believe it would be potentially misleading to make any claims beyond the UK.
We have added the following paragraph to the discussion:
This study focuses on the UK, where salt-reduction initiatives and product-specific benchmarks have been in place for many years [UK Benchmark]. Although our sample is small and not designed to be representative of all UK foods, it illustrates the degree of compositional variability that is likely to occur more widely, given that production processes are broadly similar across settings. A larger, country-specific investigation would be required to assess compliance with international WHO benchmarks [WHO Benchmark].
